# Development of a target identification approach using native mass spectrometry

**DOI:** 10.1038/s41598-021-81859-4

**Published:** 2021-01-27

**Authors:** Miaomiao Liu, Wesley C. Van Voorhis, Ronald J. Quinn

**Affiliations:** 1 Griffith Institute for Drug Discovery, Griffith University, Brisbane, QLD 4111 Australia; 2 Department of Medicine, Center for Emerging and Re-Emerging Infectious Diseases, University of Washington, 750 Republican St, Seattle, Washington 98109-4766 USA

**Keywords:** Drug discovery, Target identification, Mass spectrometry

## Abstract

A key step in the development of new pharmaceutical drugs is the identification of the molecular target and distinguishing this from all other gene products that respond indirectly to the drug. Target identification remains a crucial process and a current bottleneck for advancing hits through the discovery pipeline. Here we report a method, that takes advantage of the specific detection of protein–ligand complexes by native mass spectrometry (MS) to probe the protein partner of a ligand in an untargeted method. The key advantage is that it uses unmodified small molecules for binding and, thereby, it does not require labelled ligands and is not limited by the chemistry required to tag the molecule. We demonstrate the use of native MS to identify known ligand–protein interactions in a protein mixture under various experimental conditions. A protein–ligand complex was successfully detected between parthenolide and thioredoxin (*Pf*Trx) in a five-protein mixture, as well as when parthenolide was mixed in a bacterial cell lysate spiked with *Pf*Trx. We provide preliminary data that native MS could be used to identify binding targets for any small molecule.

## Introduction

The use of bioactive small molecules for the treatment of disease is an integral part of human culture. Despite remarkable achievements in genomics, transcriptomics, proteomics, and metabolomics, a major bottleneck in the drug discovery process remains target identification. Over the years, several new target identification strategies have been developed and the number of successful examples steadily grows. However, many target identification strategies require the synthesis of a probe molecule that is time consuming, resource intensive and there is no guarantee that a probe, that retains its target binding capability, can be found either due to the complexity of the binding or synthetic limitations. Significant advantages accrue to non-probe methods that can detect a protein–ligand interaction in a complex cell lysate.

**Figure 1:**
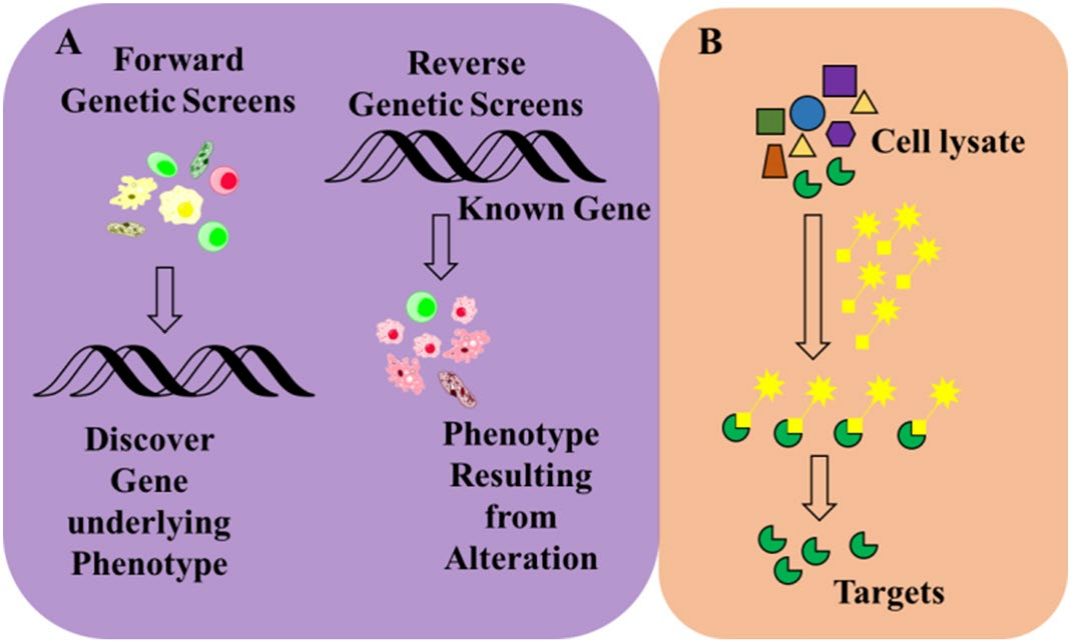
Basic workflow of target identification: (**A**) Genetic approach; (**B**) Biochemical approach.

In general, target identification strategies can be classified into two major directions: genetic approaches and biochemical approaches (Fig. 1). The genetic approaches (Fig. 1A) include forward and reverse genetics. Forward genetics is the identification of a phenotype followed by determination of the gene underlying the phenotype^1–3^. Forward genetic screens measure cellular function without identifying the relevant targets or signalling pathways and allow the discovery of new therapeutic targets^4^. As such, phenotypic assays require a subsequent effort to discover the molecular targets of bioactive small molecules, which can be a very complex process. One of the most traditional genetics approaches to target identification is the identification of a gene causing a resistance phenotype. Identifying and characterizing drug-resistant clones is relatively simple, but it is typically limited to microbial systems and may not always succeed as there are multiple ways resistance can appear to a drug molecule^5^. Examples are the identification of the targets of rifampicin, which targets the β-subunit of RNA polymerase and novobiocin, which targets gyrB and parE^6,7^. Reverse genetics is a widely-used approach in which a specific gene of interest is mutated or deleted followed by a broad search for the resultant phenotype^8,9^. In this approach, subsequent target validation is essential, but is often very time-consuming, and involves demonstrating the relevance of the protein for a particular biological pathway^10^. Such an impact needs to be confirmed in cells or animals by demonstrating that target inhibition/activation correlates with the phenotypes^11^.

Biochemical strategies (Fig. 1B) generally involve isolating the proteins that directly bind to the molecule. Affinity-based proteomics (“pulldown”) is one of the most widely applied target identification approaches. It involves a modified or labelled compound “bait” to identify the interacted target proteins, which usually require large amounts of compounds. The major challenges of this method are (1) most of the low molecular weight compounds may not have an appropriate modification site available; (2) the function of the modified molecule may be different from the unmodified compounds. This approach is particularly not suitable for natural products because of limited compound supply and relative poor structure–activity relationship information available^12,13^. Recently, several studies reported label-free strategies that directly detect the thermodynamic stability changes of the target protein upon ligand binding^14–16^. Some examples are cellular thermal shift analysis-mass spectrometry (CETSA-MS)^17^, drug affinity responsive target stability (DARTS)^16^, stability of proteins from rates of oxidation (SPROX)^18^ and yeast haploinsufficiency (HIP), and homozygous (HOP) profiling^19^. These approaches have great potential and nicely complement traditional affinity methods, however, most of them are limited to specific proteins. Another limitation is that these methods are more suited for high-affinity ligands that bind relatively abundant target proteins.

Mass spectrometry (MS) can be used to find a ligand that binds to a protein by identification of the ligand. These methods are based on MS identification of the ligand following affinity-based capture and/or release and provide an indirect observation. MS advantages include rapidity, high sensitivity and specificity. In particular, it does not require labelling on either proteins or the ligands, and thus widens the application area for these methods. Applications of MS for studying the interactions between small molecules and biological macromolecules have been previously reviewed^20–22^, Specific examples are affinity selection mass spectrometry (ASMS) and pulsed ultrafiltration-mass spectrometry (PUF-MS)^23,24^. They have been developed and applied as powerful tools to conduct high throughput screening on large compound libraries or natural product extracts for biologically active compounds against drug targets^25,26^.

**Figure 2:**
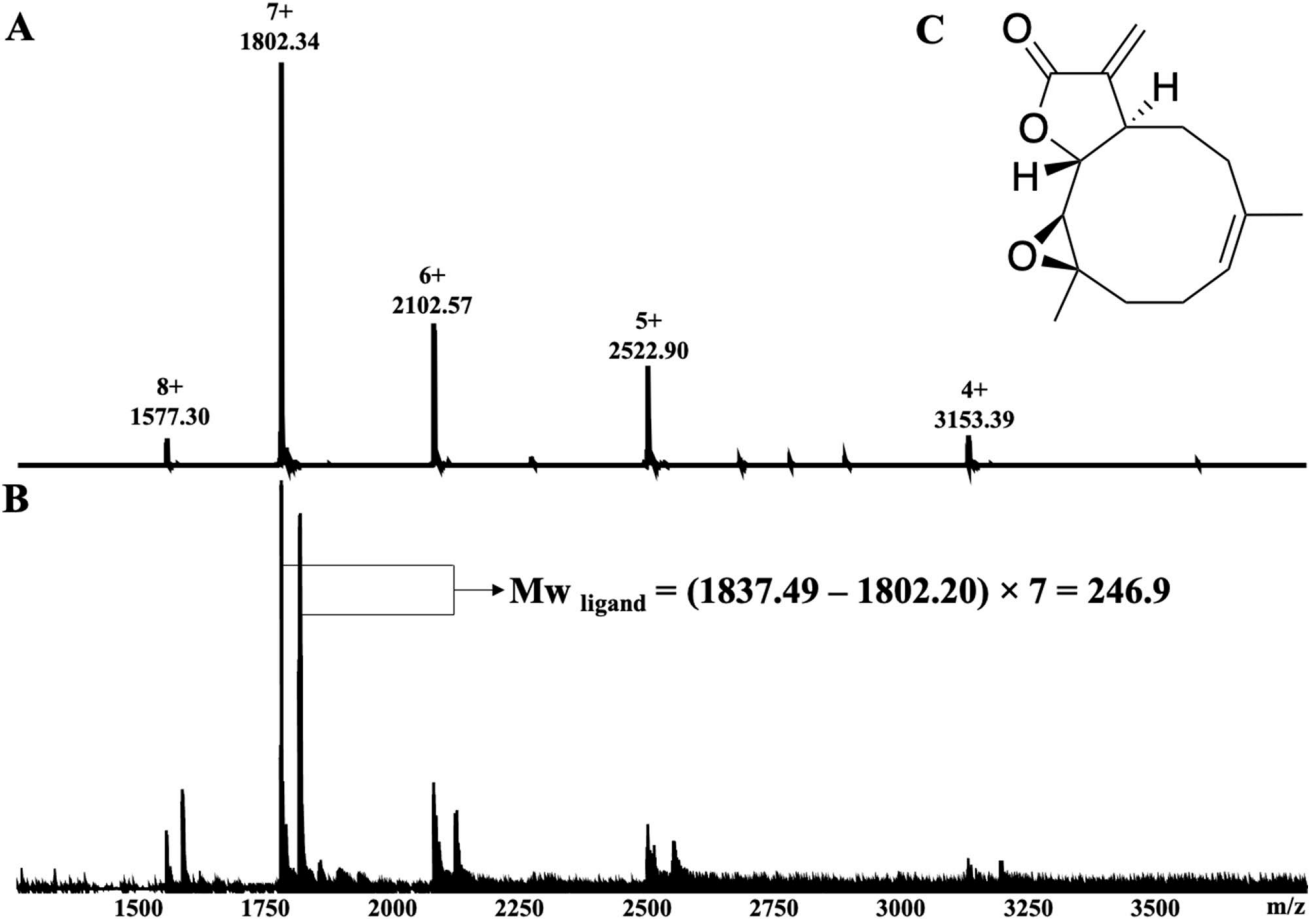
Typical native MS showing major 7 + charge state together with the 8 + and 6 + charge state species: (**A**) thioredoxin (*Pf*Trx); (**B**) *Pf*Trx with parthenolide; (**C**) chemical structure of parthenolide.

Direct observation of protein–ligand complexes involves detection of the protein–ligand complexes without disrupting the non-covalent interactions by the use of soft ionization technologies, such as electrospray mass spectrometry (ESI–MS) and matrix assisted laser desorption ionization mass spectrometry (MALDI-MS). The major advantage of native ESI–MS is the possibility to directly investigate protein–ligand interactions under non-denaturing conditions. Both non-covalent and covalent protein–ligand complexes can be detected by native mass spectrometry^27,28^. Ligands are identified by observation of mass-to-charge ratio shifts and the molecular weight of the ligand can be determined by calculation of the mass differences between unbound protein and protein–ligand complex (Fig. 2). We recently reported the use of native MS with high-resolution electrospray ionization Fourier transform ion cyclotron resonance mass spectrometry (ESI-FT-ICR-MS) for direct protein–ligand detection between individual proteins and compound libraries or natural product extracts^29–33^. Comprehensive instrumental ESI source condition optimizations have been conducted to maximize the relative ionization efficiency of the protein–ligand complex over individual free protein^27,28,34^. The technology is robust, including allowing the detection of low affinity fragments (up to 1 mM) for fragment-based drug discovery screenings^35,36^. Compared with native ESI–MS, MALDI-MS has been used much less in the studies of protein–ligand interactions. MALDI-MS has been more widely applied to study the spatial distribution characteristics of small molecules in vivo^37,38^. One study has shown direct detection of the protein–ligand complex of the bovine serum albumin and four tannin β-1, 2, 3, 4, 6-5-O-galacyl-d-glucose (PGG) molecules by MALDI-MS^39^. Surface based MS approaches have been used for direct protein–ligand binding studies. The liquid sample desorption electrospray ionization–mass spectrometry (DESI-MS) has been successfully applied to detect intact protein−ligand complexes formed between ribonuclease A with cytidine nucleotide ligands and lysozyme with acetyl chitose ligands^40,41^. The liquid extraction surface analysis–mass spectrometry (LESA-MS) has been widely used for simultaneous analysis of native protein structure and spatial distribution within thin tissue sections, showing advantages over DESI-MS by the ability of detecting intact proteins up to ~ 800 kDa. Recently, several studies reported the use of native LESA-MS to detect non-covalent protein complexes^42–45^.

In the current work, we explore the possibility to use a ligand to probe the protein partner using native MS with an objective to complement screening of compound libraries against a protein target. We investigated interactions between a ligand and a mixture of five proteins and spiking experiments in a cell lysate using native MS. A series of experimental conditions including ammonium acetate concentration, pH, and ESI parameters were used to explore effects of the selected instrument and solution parameters on protein–ligand interactions. This study demonstrated the potential of native MS to probe the protein partner of a ligand in a group of proteins.

## Results and discussion

### Protein selection

We have previously reported native MS screening in fragment-based drug discovery on cloned and purified malarial proteins^35^. We reported 31 proteins worthy of further investigation as anti-plasmodial targets as these 31 proteins had 79 low-molecular-weight natural product hits with in vitro antimalarial activity. Five malarial proteins and one fragment from the previous study were selected for this study.

Adenosine deaminase from *Plasmodium vivax* (*Pv*ADA) is important in purine metabolism by deaminating adenosine and deoxyadenosine to form the respective inosines^46^. *Pv*ADA has also been reported to play critical roles in purine salvage pathways in microorganisms and *P. falciparum*^47^. Studies have shown that inhibition of *Pv*ADA resulted in parasite death, highlighting the possibility of *Pv*ADA as a potential drug target^48^. A serial of known *Pv*ADA inhibitors have been identified, e.g. coformycin and 2′-deoxycoformycin (also known as pentostatin)^49,50^. *Pf*dUTPase catalyzes the hydrolysis of dUTP to dUMP and supplies the dUMP substrate for dTTP synthesis, which is essential in both eukaryotes and prokaryotes^51^. Sequence similarity between *Pf*dUTPase and its human ortholog is only 28.4%, making it a suitable drug target^52^. Thioredoxin (*Pf*Trx) belongs to the thioredoxin superfamily comprising the dithiol-containing redox proteins^53^. It has been reported that *Pf*Trx acts as an important component of malaria parasite protein secretion machinery^54^. Inhibition of *Pf*Trx function by inhibitors made parasite unable to secrete pathogenic proteins into hosts, providing a new drug target for anti-malarial drug development^55^. Ubiquitin conjugated enzyme (*P. falciparum*) is a key member involved in ubiquitylation process and plays significant role in the survival and propagation of *P. falciparum*^56^*.* Throughout its life cycle, ubiquitylation has the central in cell differentiation, which is required in parasite survival, making ubiquitylation enzymes as potential targets in drug discovery^57^. The last protein serine/threonine protein kinase *Pv*NEK4 belonging to the NIMA-related protein kinases (Neks), plays central roles in protein phosphorylation^58^. It has been shown that *Pv*NEK4 is essential in zygote-to-ookinete transformation, by regulating DNA replication that precedes meiosis in *Plasmodium*^59^.

The five proteins were analyzed individually and native MS spectra are shown in Figure S1. *Pv*ADA, displayed a major 14 + (*m/z* 3059) charge state together with a 15 + (*m/z* 2855) and 13 + (*m/z* 3294) charge state species, giving the molecular weight as 42,812 Da. *Pf*dUTPase, showed a major charge state 9 + (*m/z* 2261) along with 10 + (*m/z* 2034) and 8 + (*m/z* 2543) charge states giving a molecular weight of 20,340 Da. *Pf*Trx showed a major 7 + (*m/z* 1802) charge state as well as charge states 8 + (*m/z* 1576), 6 + (*m/z* 2102) and 5 + (*m/z* 2522), giving a molecular weight of 12,607 Da. Ubiquitin conjugated enzyme (*P. falciparum*), with molecular weight 23,770 Da showed a major charge state 10 + (*m/z* 2378) together with 11 + (*m/z* 2162) and 9 + (*m/z* 2649) species. Serine/threonine protein kinase *Pv*NEK4, showed a major charge state 14 + (*m/z* 2649) with other charge states 15 + (*m/z* 2472) and 13 + (*m/z* 2853), giving a molecular weight as 37,072 Da.

### Native MS of combined 5 proteins

The five proteins were then mixed together and investigated under twelve concentration / pH conditions and five MS instrument conditions for effects on protein intensities in the protein mixture.

### Assay conditions

**Figure 3:**
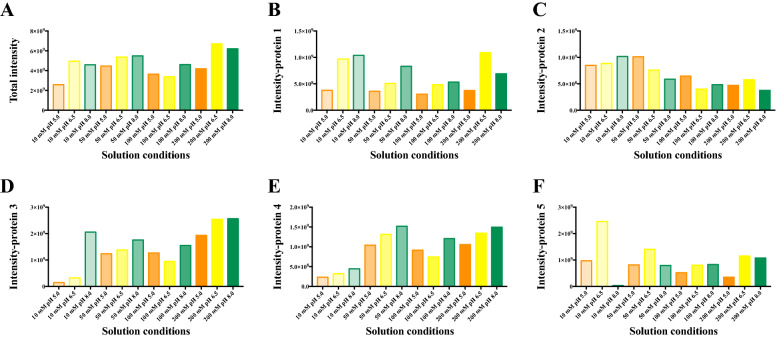
Native MS protein signal intensities of the protein mixture under different ammonium acetate concentration/pH conditions (instrument condition: skimmer 30 V). (**A**) total protein signals; (**B**) *Pv*ADA signals, (**C**) *Pf*dUTPase signals, (**D**) *Pf*Trx signals, (**E**) Ubiquitin conjugated enzyme (*P. falciparum*) signals, (**F**) *Pv*NEK4 signals.

Native MS involves the transfer of intact proteins from solution into the gas phase. Ammonium acetate is a volatile electrolyte that can mimic the solvation properties experienced by proteins under physiological conditions and, therefore, has been widely used in native MS studies^60^. Protein stability is usually reduced in the absence of dissolved salts, and ammonium acetate can serve as a stabilizing background electrolyte by providing the necessary ionic strength and pH for proteins and protein complexes to fold and assemble^61^. In this study, we investigated effects of different concentrations and pH values of ammonium acetate on the total MS intensities of the protein mixture (Fig. 3). It has been reported that concentrations of non-volatile salts greater than 100 mM can lead to broad, unresolved peaks in a native mass spectrum^62^. However, in our study, we found amongst the four investigated concentrations of ammonium acetate (10 mM, 50 mM, 100 mM, and 200 mM), that the highest protein total signal intensities were observed in 200 mM ammonium acetate, showing an increase of about 1.37 times in signal intensities of the protein mixture compared to 100 mM ammonium acetate under the same instrument tuning condition (Fig. 3A). Different proteins behave very differently in the protein mixture under different ammonium acetate concentration/pH conditions (Fig. 3B–F). For example, the MS signals of *Pf*dUTPase showed a decreasing trend as the concentration increased, while proteins *Pf*Trx and ubiquitin conjugated enzyme (*P. falciparum*) displayed an opposite MS signal intensity change. Effects of different pH (5.0, 6.5, and 8.0) on native MS spectra of the protein mixture were investigated from solutions with the same nominal ionic strength. In an effort to mimic the intracellular environment, classic biochemical experiments are generally conducted in neutral aqueous solutions with pH around 6.5–7.0. This is consistent with the result of total protein intensities in the protein mixture (Fig. 3A), pH 6.5 ammonium acetate giving the highest protein signals compared to pH 8.0 and 5.0 ammonium acetate. However, when only considering signals of individual proteins in the protein mixture, only *Pv*NEK4 showed significant higher signal intensities at pH 6.5 (Fig. 3F). Proteins *Pv*ADA, *Pf*Trx and ubiquitin conjugated enzyme (*P. falciparum*) were found to give higher MS signals at pH 8.0, and for *Pf*dUTPase, the highest protein signals were observed at pH 5.0 (Fig. 3C). It is clear that different protein signals can be observed by changing ammonium acetate concentrations or pH values, suggesting an effective way to observe proteins in a complex cell lysate.

### ESI–MS conditions

We have reported previous studies of systematic optimisation of MS instrument conditions to detect proteins in their native states and to detect both non-covalent and covalent complexes with small molecules^27,30^. The design of this study relies on our previous experiments, three parameters skimmer, CID (collision induced dissociation) and ISCID (in-source collision induced dissociation) were selected to investigate effects on protein signals in the 5-protein mixture (Table 1). Skimmer is an important parameter that plays a role in the level of collisional activation occurring along the path from the atmospheric region to the high vacuum region and influences the intensity of signals^63^. CID is a technique for fragmenting ions in the gas phase, whereby ions are accelerated by an electrical potential and allowed to collide with neutral gas molecules such as argon or xenon^64^. ISCID is a type of CID in which ions are fragmented in the source region of the mass spectrometer^65^.

**Table 1:** List of selected instrument conditions.

Instrument conditions	Skimmer (V)	CID (V)	ISCID (V)
1	30	0	0
2	90	0	0
3	30	5	0
4	30	0	60
5	30	5	60

**Figure 4:**
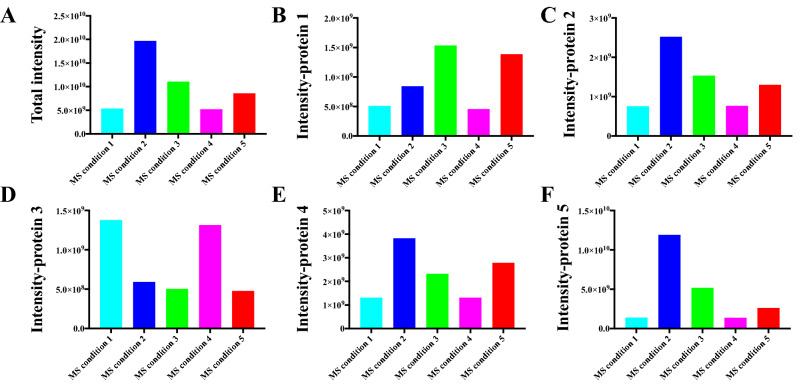
Native MS protein signal intensities of the protein mixture under different ESI–MS conditions (50 mM ammonium acetate, pH 6.5). (**A**) total protein signals; (**B**) *Pv*ADA signals, (**C**) *Pf*dUTPase signals, (**D**) *Pf*Trx signals, (**E**) Ubiquitin conjugated enzyme (*P. falciparum*) signals, (**F**) *Pv*NEK4 signals.

Two levels were chosen in the following ranges: skimmer 30 V and 90 V, CID voltage 0 V and 5 V, ISCID voltage 0 V and 60 V. Among the five investigated ESI–MS conditions, the highest total protein signals were observed under condition 2 (Fig. 4A). A higher skimmer in condition 2 compared with condition 1 resulted in a 3.7 times protein signal increase. A similar effect was found by increasing CID voltage from 0 to 5 V (condition 3), which led to 2.1 times protein signal improvement. However, changing ISCID voltage from 0 to 60 V did not contribute to protein signal intensities, inversely, a 1.6 × 10^8^ protein signal intensity drop was observed under condition 4. Condition 5 with both CID voltage of 5 V and ISCID voltage of 60 V did not result in higher protein intensities compared with condition 3 (CID voltage 5 V, ISCID voltage 0 V). It was observed that some factors have a differential influence on individual proteins in the protein mixture (Fig. 4B–F). Proteins *Pf*dUTPase, ubiquitin conjugated enzyme (*P. falciparum*) and *Pv*NEK4 showed similar trends to the total protein signals under different ESI–MS conditions, highest and lowest intensities found in conditions 2 and 1 (*Pf*dUTPase) or 4 (proteins ubiquitin conjugated enzyme and *Pv*NEK4), respectively. Higher protein intensities were observed for *Pv*ADA in conditions 3 and 5, however, condition 4 only kept 30% of those signals under condition 3, indicating ion fragmentation has a critical role on this specific protein (Fig. 4B). More surprisingly, the maximum absolute intensity of *Pf*Trx was found when applying conditions 1 and 4, changing to higher skimmer or CID voltage only decreasing protein intensities (Fig. 4D). It supports the hypothesis that different proteins in a cell lysate could be detected by using different MS instrument conditions.

**Figure 5:**
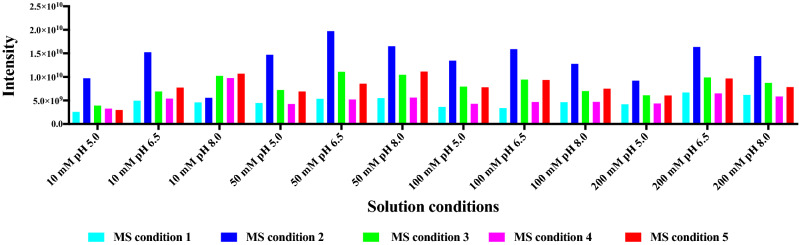
Native MS protein signal intensities of the protein mixture under different ammonium acetate concentration/pH and instrument conditions.

Twelve concentration/pH conditions and five MS instrument conditions were investigated together on effects on protein intensities in the protein mixture (Fig. 5). Skimmer seems to be the most important factor on affecting protein intensities. The maximum and minimum absolute intensities of total protein signals were mostly observed from condition 2 (skimmer 90 V) and condition 1 (skimmer 30 V) for all ammonium acetate concentration/pH conditions except for 10 mM ammonium acetate at pH 8.0, in which the maximum protein intensity appeared under instrument condition 5 (skimmer 30 V, CID 5 V and ISCID 60 V). Individual protein intensities under various ammonium acetate concentration/pH and MS instrument conditions were also investigated. As expected, the optimum condition for each protein is different, *Pv*ADA in 10 mM, pH 8.0 ammonium acetate under MS condition 3, *Pf*dUTPase in 100 mM, pH 5.0 under MS condition 2, *Pf*Trx in 10 mM, pH 8.0 under MS condition 4, ubiquitin conjugated enzyme (*P. falciparum*) in 50 mM, pH 8.0 under MS condition 2, and *Pv*NEK4 in 50 mM, pH 6.5 under MS condition 2 (Figure S2–S6). Protein signal intensities of *Pf*Trx under different buffer conditions and MS instrument conditions were also evaluated (Figure S7). Similar intensity changing patterns were observed between *Pf*Trx itself and in the protein mixture, suggesting similar protein behaviours when mixed with other proteins.

### Conditions to preserve noncovalent complexes in the protein mixture

**Figure 6:**
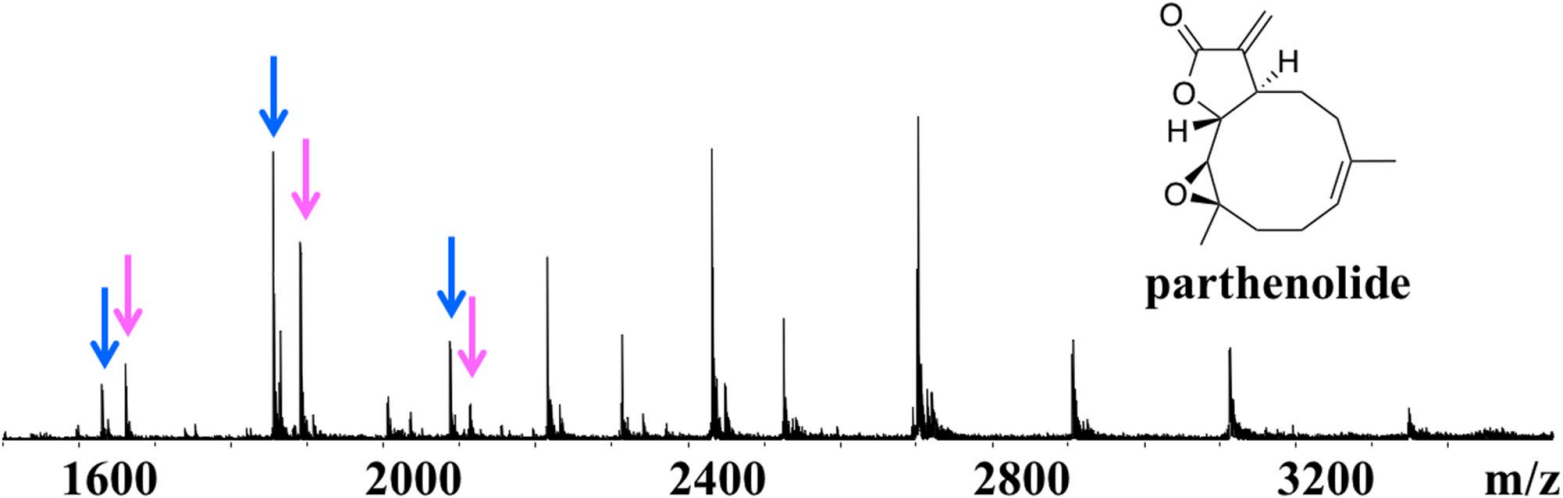
Native MS spectra of protein–ligand complex formed between the 5-protein mixture and ligand parthenolide. Blue arrows: unbound *Pf*Trx, magenta arrow: protein–ligand complex.

The native MS target ID approach relies on detection of non-covalent protein–ligand interaction between small molecule and target protein(s) from a protein mixture. Parthenolide was previously reported to bind to *Pf*Trx (Fig. 2)^35^. We examined interaction between parthenolide and the 5-protein mixture. The same protein–ligand complex was clearly observed under native MS condition (Fig. 6), suggesting a high potential of the native MS target ID approach to capture protein–ligand complexes from more complex matrixes. Non-covalent complexes, however, might be disrupted under harsh experimental conditions. Parameters such as concentration, pH or instrument conditions skimmer, CID and ISCID play critical roles in the intensity of complex signals, therefore have been explored in this study.

**Figure 7:**
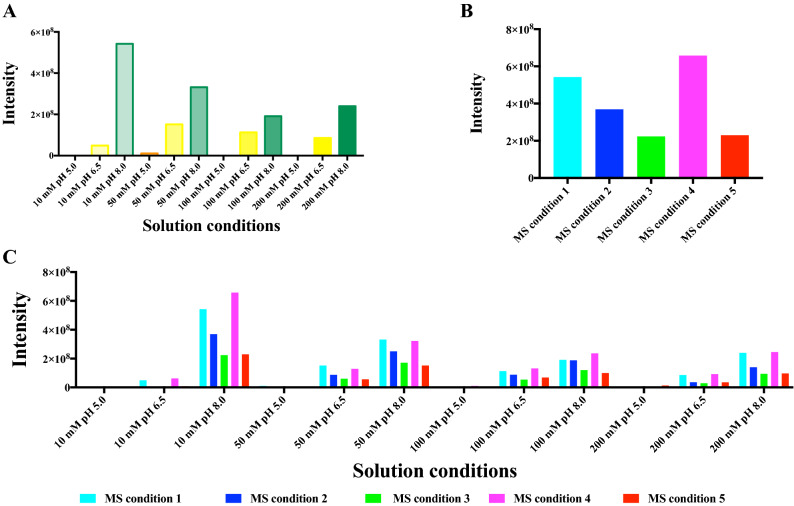
Protein–ligand complex detected between the protein mixture and ligand parthenolide under different experimental conditions. (**A**) twelve ammonium acetate concentration/pH conditions (instrument condition: skimmer 30 V), (**B**) five MS instrument conditions (10 mM ammonium acetate, pH 8.0), (**C**) sixty conditions.

The previous twelve ammonium acetate conditions (4 concentrations × 3 pH levels) were evaluated for abilities to preserve non-covalent complex. The pH has a greater influence on the formation of the complex compared to concentration (Fig. 7A). No protein–ligand complex was formed when using acidic conditions (pH 5.0), while increasing the pH to 6.5 and further to 8.0, allowed detection of protein–ligand complex signals. The optimal condition for protein–ligand complex generation was 10 mM, pH 8.0 ammonium acetate solution. Regarding the MS instrument conditions, a mild condition, such as condition 1 (skimmer 30 V) detected higher protein–ligand complex signals than harsh conditions 2 (skimmer 90 V), 3 (skimmer 30 V, CID 5 V) and 5 (skimmer 30 V, CID 5 V, ISCID 60 V) (Fig. 7B). Exception appears with condition 4 (skimmer 30 V, ISCID 60 V), which resulted in the maximum protein–ligand complex intensity among all of the tested conditions. It has been reported that increasing the voltage of ISCID could help better desolvate the complex without dissociating it, resulting in narrower peak widths, smaller peak fluctuations and therefore higher signal intensities^66^. Figure 7C shows details of protein–ligand complex detected under the 60 tested conditions. Experiments were repeated to explore effects of different conditions on protein–ligand complex formed between parthenolide and *Pf*Trx (Figure S8). In general, a similar pattern was observed in detection of protein–ligand complex with a pure protein or with a protein mixture under different experimental conditions, providing strong evidence that detection of protein–ligand complex by native MS from a protein mixture can describe the same nature of interactions between the ligand with its target protein. Therefore, it was also confirmed that purified proteins are not required in the proposed native MS target ID approach.

### Interactions of ligand and protein in different matrixes

**Figure 8:**
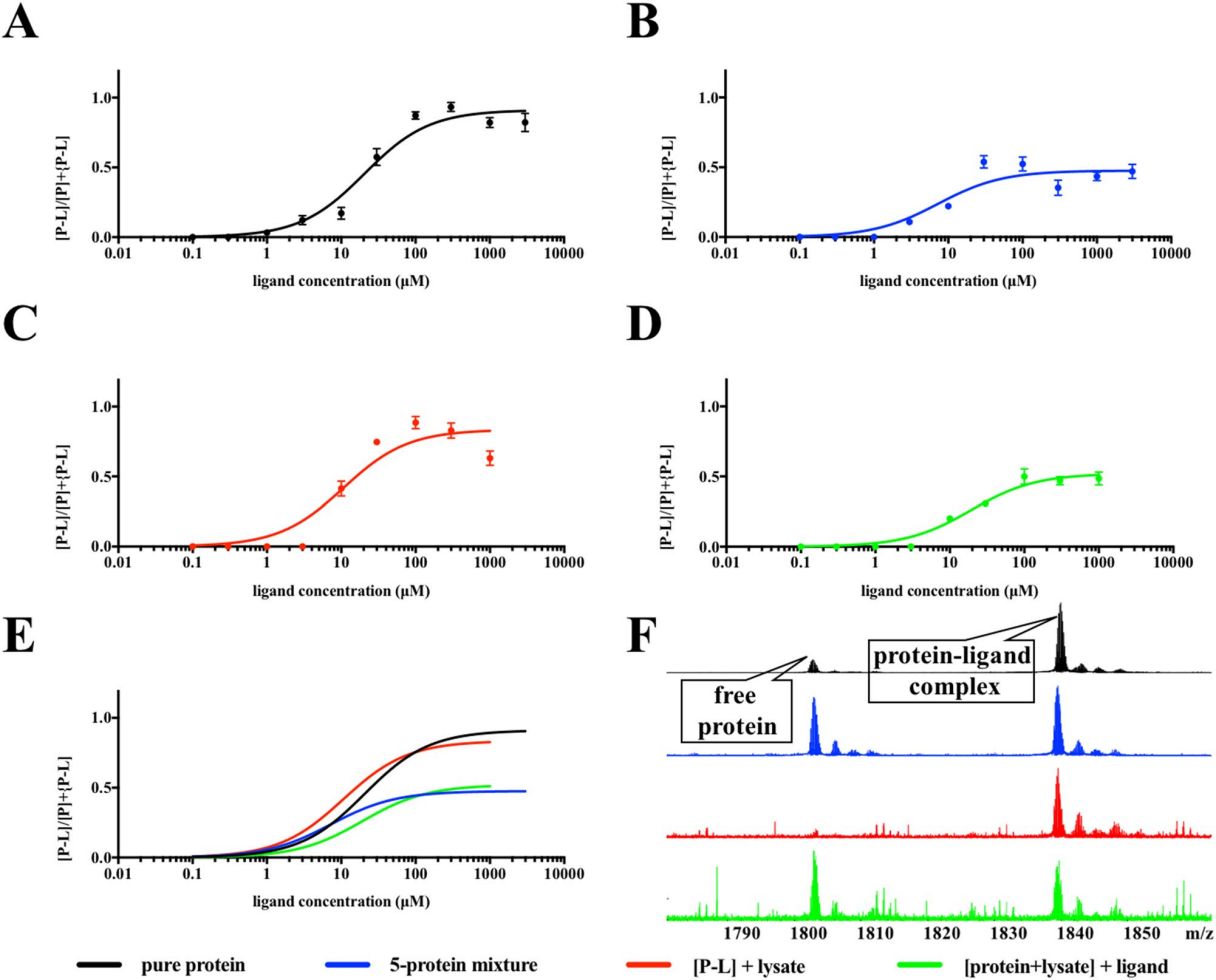
(**A**) Plot of [P-L]/[P] + [P-L] versus ligand concentration for the titration of *Pf*Trx with parthenolide. (**B**) Plot of [P-L]/[P] + [P-L] versus ligand concentration for the titration of *Pf*Trx in the 5-protein mixture with parthenolide. (**C**) Plot of [P-L]/[P] + [P-L] versus ligand concentration for the titration of cell lysate with pre-incubated *Pf*Trx and parthenolide. (**D**) Plot of [P-L]/[P] + [P-L] versus ligand concentration for the titration of cell lysate added with *Pf*Trx and parthenolide. (**E**) Comparison of the four titration curves. Symbols and error bars were removed from this plot. (**F**) Stacked MS spectra acquired from 100 μM parthenolide and 9 μM protein *Pf*Trx in different matrixes.

One of the critical questions in the proposed native MS target ID is to determine the affinity required to maintain the protein–ligand complexes in different matrixes. Based on the evaluation results shown above, 10 mM, pH 8.0 ammonium acetate with MS instrument condition 1 (skimmer 30 V) was selected as the optimum tuning condition. Four matrixes with different levels of protein background complexity were evaluated (Fig. 8). Firstly, a titration experiment was conducted using a constant concentration (9 μM) of *Pf*Trx and titrating the parthenolide (Fig. 8A). Among the ten increasing concentrations of parthenolide (0.1–3000 μM), protein–ligand complex started to be detected with 1 μM addition of ligand, and 30 μM of ligand increased the radio between protein–ligand complex and the entire protein (protein–ligand complex + free protein) in the solution to around 0.57. When the ligand concentration was further increased to 100—3000 μM, the intensity of the protein–ligand complex reached a plateau with maximum protein–ligand to protein ratio of 0.97. Secondly, the titration experiment was repeated with parthenolide and the 5-protein mixture, in which each protein concentration was kept at a constant 9 μM (Fig. 8B). The minimum ligand required for protein–ligand complex detection from the 5-protein mixture was 3 μM, and the maximum protein–ligand to protein radio at 0.58 was reached with 100 μM of ligand addition. Further increasing of ligand only led to protein–ligand complex intensity loss. Around 3 times higher ligand concentrations were required to detect the minimum protein–ligand complex from a 5-protein mixture compared to the pure protein matrix. A bacterial cell lysate was used as a more complicated matrix in a target ID study. Two different titration experiments were conducted. Firstly, parthenolide, at increasing concentrations, was pre-incubated with *Pf*Trx (9 μM) and then added to the cell lysate followed by native MS analysis (Fig. 8C). Similar to the results from the pure protein experiment, the intensity of the protein–ligand complex reached a plateau with 30 μM ligand and the maximum protein–ligand to protein radio was around 0.93. However, protein–ligand complex was only detected with a ligand concentration of at least 10 μM, which is 10 times higher than the pure protein experiment. Secondly, 9 μM protein was initially mixed with the cell lysate and parthenolide, at increasing concentrations, was then added to the spiked cell lysate (Fig. 8D). The minimum ligand concentration required was found to be 10 μM, the same value when the protein–ligand mixture was added to the cell lysate. However, the maximum protein–ligand to protein radio only reached to 0.43, showing the influence from the complicated background signals. A comparison of native MS spectra acquired from each titration experiment at the same ligand and protein concentration are shown in Fig. 8F. This provided the detection limit of a protein–ligand complex in a cell lysate.

We demonstrated the use of native MS method to investigate a known ligand–protein interaction in a protein mixture under various experimental conditions, including different ammonium acetate concentration/pH conditions and different MS instrumental parameters. Five malarial proteins *Pv*ADA, *Pf*dUTPase, *Pf*Trx, ubiquitin conjugated enzyme (*P. falciparum*) and *Pv*NEK4 were mixed together to mimic the complex cell lysate environment. Although there was some suppression of signal compared to the individual protein, all five individual proteins were visible with reasonable intensities. Amongst the 12 investigated conditions of ammonium acetate (10 mM, 50 mM, 100 mM and 200 mM under pH 5.0, 6.5 and 8.0), we found the highest total protein signal intensities appeared at 200 mM, pH 6.5. Compared to individual protein signals, the protein mixture behaved differently. The highest total protein signals in the protein mixture were found when applying a higher skimmer of 90 V (condition 2), which also resulted in the highest protein signals of individual proteins *Pf*dUTPase, ubiquitin conjugated enzyme (*P. falciparum*) and *Pv*NEK4. Proteins *Pv*ADA and *Pf*Trx behaved differently under the selected ESI–MS conditions, suggesting that different proteins in a cell lysate could be detected by using different MS instrument conditions.

We have successfully confirmed the interaction between parthenolide and *Pf*Trx in the protein mixture, as well as when parthenolide was mixed in a bacterial cell lysate spiked with *Pf*Trx. A total number of four matrixes with different levels of protein background complexity were evaluated, including *Pf*Trx (matrix 1), the 5-protein mixture (matrix 2) and 2 matrixes with addition of a bacterial cell lysate (matrix 3 and 4). The protein–ligand complex was successfully detected from each matrix, supporting the potential of the native MS approach to study protein–ligand interactions in a background of non-target proteins. Less protein–ligand complex was formed or detected in matrixes 2 and 4 under the same experimental condition. Secondly, protein–ligand complex detection limits were gradually decreased from matrixes 1 to 4, suggesting higher concentration of ligand would be required when detecting protein–ligand interactions in complicated protein matrixes. Similar levels of protein–ligand complex was detected with ligand addition to protein (matrix 1) and pre-incubated protein–ligand to cell lysate (matrix 3), suggesting that effects by non-target protein are minimal.

A key challenge in the pre-clinical development of small molecule drugs, which comprise most of today’s medicines, is the identification of the molecular target(s) underlying the therapeutic effects. Most approved drug that are administered to patients consist of a single active chemical entity. As modification of a chemical entity changes its biological properties, significant chemical resources are required to attach chemical tags/fluorophores and then to confirm that the biological response is identical, or similar to the unmodified compound. With the preliminary data presented in this paper, we propose a strategy of using a native MS approach to probe the protein partner of a ligand or target identification of bioactive molecules. Soluble proteins from a cell lysate can be directly probed by a potential drug or bioactive molecule. The protein target can be identified by the appearance of new peaks corresponding to a protein–ligand complex. These two peaks (protein–ligand peak and the endogenous target protein peak) should have the same charge state, and the m/z relationship between the complex peaks and the protein peaks is: m/z (protein) = m/z (complex) – MW (ligand)/charge state. As the molecular mass of the ligand is known, the associated protein peak can be identified. Either database identification or de novo sequencing would allow the target to be identified. The key advantage is that because it does not require labelled ligands and instead uses unmodified small molecules for binding, it is not limited by chemistry (to synthesis derivatives) and can potentially be used to identify binding targets for any small molecule. Unlike cell-based methods, native MS is completely independent of any effects of the drug on the system, and is therefore compatible with any mechanism of action, making it useful for any small molecule of interest. Native MS can be performed by using any cell or tissue type from any organism and is thus not limited by the availability and coverage of knockout (or knockdown) libraries and genome arrays for model organisms.

Potential limitations of the native MS approach cannot be ignored. Firstly, the binding affinity of the drug to its target may be a limiting factor. The complexity of cell lysates may be another potential risk however prior cell lysate fractionation is being investigated in on-going development of this method.

Even in this molecular era of drug discovery, there remain new investigational drugs whose molecular targets are unclear, restricting their optimisation and broad use in disease. The ability of the proposed method to rapidly identify molecular targets, supports the idea that this method will be useful in target identification for drugs that emerge from phenotype methods.

## Methods

### Proteins

Five proteins from *P. falciparum* were expressed in *Escherichia coli*. In general, genes were cloned into expression vectors that enabled tagging of the corresponding proteins with an N-terminal 6-histidine tag, as previously described^67,68^. Proteins were purified using a nickel column (immobilized-metal affinity chromatography, IMAC), followed by size-exclusion chromatography, concentrated, flash frozen, stored at − 80 °C, and shipped on dry ice.

### Ligand

Parthenolide was previously isolated in our laboratory^69^.

### Protein preparation

Individual protein experiments: each protein was exchanged into ammonium acetate under selected concentrations (10 mM, 50 mM, 100 mM and 200 mM) and pH values (5.0, 6.5 and 8.0) using size exclusion chromatography (Nalgene NAP-5 size G25, GE Healthcare) prior to native-MS analysis. Final concentration of each protein was 10 μM.

Protein mixture experiments: five proteins were mixed and followed by buffer-exchanged into ammonium acetate under selected concentrations (10 mM, 50 mM, 100 mM and 200 mM) and pH values (5.0, 6.5 and 8.0) using size exclusion chromatography prior to native − MS analysis. Final concentration of each protein in the mixture was 10 μM.

### Instrument control and acquisition

Experiments were performed on a Bruker SolariX XR 12 T Fourier transform ion cyclotron resonance mass spectrometry (ESI-FT-ICR-MS) (Bruker Daltonics Inc., Billerica, MA) equipped with an automated chip-based nano-electrospray system (TriVersa NanoMate, Advion Biosciences, Ithaca, NY, USA). Individual proteins or protein mixture were injected to MRMS to evaluate effects of ammonium acetate concentration/pH and instrument conditions on protein signal intensities. For each protein–ligand interaction experiment, 1 μL of ligand parthenolide at 1 mM in methanol was incubated with 9 μL proteins (protein *Pf*Trx or protein mixture) for 30 min to 1 h at room temperature and analyzed by FTMS. Different ammonium acetate concentration/pH and instrument parameters were tested to evaluate effects on protein−ligand complex signal intensities. Mass spectra were recorded in positive ion and profile modes with a mass range from 50 to 6000 m*/z*. Each spectrum was a sum of 16 transients (scans) composed of 1 M data points. All aspects of pulse sequence control and data acquisition were controlled by Solarix control software in a Windows operating system.

### Bacterial cell lysate

*M. smegmatis* strain mc^2^155 (ATCC 70,084) was grown at 37 °C, 200 rpm for 4 days in Middle brook 7H9 broth (Becton Dickinson) supplemented with 10% OADC enrichment (Becton Dickinson) 0.05% tween-80 and 0.2% glycerol. Bacterial pellets were collected by centrifugation at 4000 rpm for 5 min and stored at − 80 °C. Cells were treated with lysis buffer (140 mM NaCl, 2.7 mM KCl, 10 mM Na_2_HPO_4_ and 1.8 mM KH_2_PO_4_, pH 7.3) on ice. After treating with cell disrupter (pressure 32 kpsi, 2 cycles), cell lysate was obtained by centrifugation at 20,000 rcf for 2 h and stored at − 80 °C.

### Titration experiments

Parthenolide solutions were prepared in DMSO by serial dilution (1 µM, 3 µM, 10 µM, 30 µM, 100 µM, 300 µM, 1 mM, 3 mM, 10 mM, 30 mM). Each concentration (1 µL) was added to each well of a V-plate microtiter plate (BioCentrix, Carlsbad, CA, USA). Then DMSO in each well was dried off using a Freeze dryer (Christ, Osterode am Harz, Germany), followed by adding 1µL MeOH in each well.

Figure 8A: Protein thioredoxin (*Pf*Trx) was buffer exchanged into ammonium acetate (10 mM, pH 8.0) using size exclusion chromatography to a final concentration of 10 µM. 9 µL protein was added to each well with compound (). Samples were incubated for 30 min to 1 h under room temperature. All sample solutions were injected by fully automated chip-based nano-electrospray. Instrument condition 1 (skimmer 30 V, CID 0, ISCID 0) was applied as the optimum tuning condition. The experiment was performed in triplicate.

Figure 8B: 5-protein-mixture were mixed and followed by buffer-exchanged into ammonium acetate (10 mM, pH 8.0) using size exclusion chromatography. Final concentration of each protein in the mixture was 10 μM. 9 µL protein mixture was added to each well with compound. Samples were incubated for 30 min to 1 h under room temperature. All sample solutions were injected by fully automated chip-based nano-electrospray. Instrument condition 1 (skimmer 30 V, CID 0, ISCID 0) was applied as the optimum tuning condition. The experiment was performed in triplicate.

Figure 8C: Protein thioredoxin (*Pf*Trx) was buffer exchanged into ammonium acetate (10 mM, pH 8.0) using size exclusion chromatography to a final concentration of 20 µM. *M. smegmatis* cell lysate was buffer exchanged into ammonium acetate (10 mM, pH 8.0) using size exclusion chromatography. 9 µL protein *Pf*Trx was added to each well with compound. Samples were incubated for 30 min to 1 h under room temperature. 10 µL cell lysate was then added to each well and incubated for 30 min under room temperature. All sample solutions were injected by fully automated chip-based nano-electrospray. Instrument condition 1 (skimmer 30 V, CID 0, ISCID 0) was applied as the optimum tuning condition. The experiment was performed in triplicate.

Figure 8D: Protein thioredoxin (*Pf*Trx) was buffer exchanged into ammonium acetate (10 mM, pH 8.0) using size exclusion chromatography to a final concentration of 20 µM. *M. smegmatis* cell lysate was buffer exchanged into ammonium acetate (10 mM, pH 8.0) using size exclusion chromatography. *Pf*Trx and cell lysate were mixed at 1:1 radio. 9 µL protein and cell lysate mixture was added to each well with compound. Samples were incubated for 30 min to 1 h under room temperature. All sample solutions were injected by fully automated chip-based nano-electrospray. Instrument condition 1 (skimmer 30 V, CID 0, ISCID 0) was applied as the optimum tuning condition. The experiment was performed in triplicate.

## Supplementary Information

Supplementary Information
