# Supplementary material for: Development of a target identification approach using native mass spectrometry
Source: Sci Rep. 2021 Jan 27;11:2387. doi: 10.1038/s41598-021-81859-4 (PMC7840913; doi:10.1038/s41598-021-81859-4)
Supplement: Supplementary file 1 — Supplementary Information [file 41598_2021_81859_MOESM1_ESM.docx]

**Development of a Target Identification Approach using Native Mass Spectrometry**

Miaomiao Liu^†^, Wesley C. Van Voorhis^‡^ and Ronald J Quinn^†^*

† Griffith Institute for Drug Discovery, Griffith University, Brisbane, Queensland 4111, Australia

^‡^ Center for Emerging and Re-emerging Infectious Diseases, Department of Medicine, University of Washington, 750 Republican St., Seattle, Washington 98109-4766, United States

**CONTENTS:**

Figure S1. Native MS spectra of the 5 individual protein.

Figure S2. Native MS protein signal intensities of protein 1 (adenosine deaminase) in the protein mixture under sixty different buffer and instrument conditions.

Figure S3. Native MS protein signal intensities of protein 2 (dUTPase) in the protein mixture under sixty different buffer and instrument conditions.

Figure S4: Native MS protein signal intensities of protein 3 (thioredoxin) in the protein mixture under sixty different buffer and instrument conditions.

Figure S5: Native MS protein signal intensities of protein 4 (ubiquitin conjugated enzyme) in the protein mixture under sixty different buffer and instrument conditions.

Figure S6: Native MS protein signal intensities of protein 5 (serine/threonine protein kinase NEK4) in the protein mixture under sixty different buffer and instrument conditions.

Figures S7. Native MS protein signal intensities of pure protein thioredoxin under different experimental conditions.

Figure S8: Protein-ligand complex detected between the pure protein 3 (thioredoxin) and ligand arthenolide under different experimental conditions.


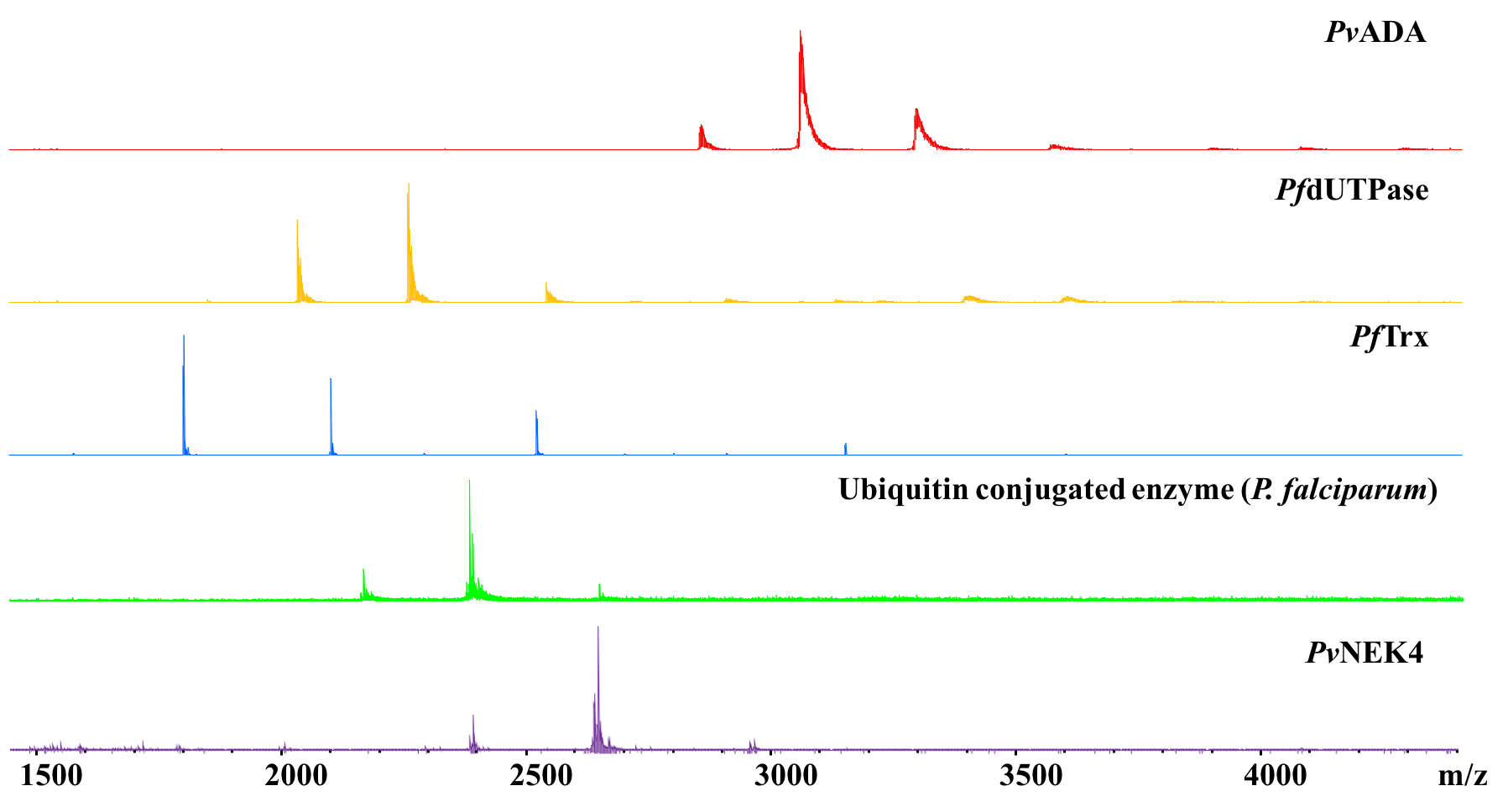


Figure S1. Native MS spectra of the 5 individual protein.

Figure S2. Native MS protein signal intensities of protein 1 (adenosine deaminase) in the protein mixture under sixty different buffer and instrument conditions.

Figure S3. Native MS protein signal intensities of protein 2 (dUTPase) in the protein mixture under sixty different buffer and instrument conditions.

Figure S4. Native MS protein signal intensities of protein 3 (thioredoxin) in the protein mixture under sixty different buffer and instrument conditions.

 Figure S5. Native MS protein signal intensities of protein 4 (ubiquitin conjugated enzyme) in the protein mixture under sixty different buffer and instrument conditions.

Figure S6. Native MS protein signal intensities of protein 5 (serine/threonine protein kinase NEK4) in the protein mixture under sixty different buffer and instrument conditions.

Figure S7. Native MS protein signal intensities of pure protein thioredoxin under different experimental conditions. A. twelve buffer conditions, B. five MS instrument conditions, C. sixty buffer and instrument conditions.

Figure S8. Protein-ligand complex detected between the pure protein 3 (thioredoxin) and ligand arthenolide under different experimental conditions. A. twelve buffer conditions, B. five MS instrument conditions, C. sixty buffer and instrument conditions.
